# Assessment of Post-Discharge Growth Pattern After Initial Growth Faltering and Its Association with the Neurodevelopment Status in Preterm Infants: A Cohort Study

**DOI:** 10.3390/nu18010125

**Published:** 2025-12-30

**Authors:** Ariadna Witte Castro, Celia Diaz Gonzalez, Susana Ares Segura, Miguel Saenz de Pipaon

**Affiliations:** Neonatology, Hospital La Paz Institute for Health Research, IdiPaz, 28046 Madrid, Spain; celia.diaz@salud.madrid.org (C.D.G.); susana.ares@salud.madrid.org (S.A.S.); miguel.saenz@salud.madrid.org (M.S.d.P.)

**Keywords:** neonatology, nutrition, outcome, prematurity

## Abstract

Background: Preterm infants are at risk of growth faltering at term age. Our primary objective is to assess post-discharge growth patterns in these infants and investigate the association between growth faltering and neurodevelopment. Methods: We divided the sample into two groups according to growth during the initial hospital stay: infants who suffered from growth faltering (GF, loss of >1 weight z-score from birth to 36 weeks postmenstrual age, n = 115) and infants who did not suffer from GF (non-growth faltering, NGF, n = 85). Results: The NFG group weight z-score was significantly lower at 36 postmenstrual ages (PMA) compared to birth (*p* < 0.001), at 1-year corrected age (CA), it was significantly higher than at birth (*p* = 0.0026), and by 2 years CA, there were no differences compared to the birth z-scores. In the GF infants’ group, statistical differences were found at all time points. At 3 and 6 months, CA GF infants were still in weight z-score values lower than −1 point compared to the birth median value. At 12 and 24 months CA, they still had not achieved birth z-score values (*p* < 0.001). In the Parent Report of Children’s Abilities-Revised (PARCA), NGF infants had a higher score in the language development scale at 2 years than GF infants (88.5 [78.5; 96.5] vs. 84.5 [69.5; 91.5], *p* = 0.03). The Bayley-III test was available for 35 infants. We found a significant difference in motor development, with a higher score in the NGF group (94 [88; 100] vs. 85 [79; 91], *p* = 0.03). Conclusions: In this cohort study, GF is associated with growth differences till 2 years CA, and with lower scores in neurodevelopment assessment.

## 1. Introduction

Appropriate growth for very preterm infants is not clearly defined. Some centers report weight z scores at 36 weeks postmenstrual age (PMA) similar to birth [1] while others reported a decrease in weight z score at 34 weeks of 0.8 compared to birth values [2]. Preterm birth can be associated with Growth Faltering (GF) [3]. This is still a common problem despite the increased knowledge and improved nutritional recommendations and clinical care [4]. The definitions of GF vary depending on the charts used [5]. GF is defined differently throughout institutions: the National Institute for Health Service in the United Kingdom (UK) defines GF as a fall in 1 centile space (0.67 z-scores) [6] in weight; the World Health Organization (WHO) defines GF as loss of 1 z-score in weight for age after 1 month or more of life from birth [7].

Many factors may influence the incidence of GF in infants, such as comorbidities [8], bronchopulmonary dysplasia (BPD) [9], late onset sepsis (LOS) [10], and Necrotizing enterocolitis (NEC) [11], sex [12], Gestational Age (GA), and nutrition [13]. In the past years, GF during hospital stay has diminished, but is still a prevalent condition, and NICU malnutrition during the first weeks of life has been associated with neurodevelopmental consequences later in life [14]. An optimal age has not been defined for these infants to recover their birth z-scores, whether before discharge or during the first year of life.

It is also crucial to ensure length gain for these GF infants to avoid imbalanced growth, developing metabolic and cardiovascular diseases associated with a higher fat percentage [15]. Length gain has been associated with neurodevelopment [14]. We compared growth until 2 years corrected age (CA) in infants who experienced GF with infants who did not experience GF, considering also length and head circumference. Additionally, we compared neurodevelopment at 2 years CA between these two groups.

## 2. Materials and Methods

### 2.1. Study Design and Participant Recruitment

In a single-center retrospective cohort study, we recorded data from a cohort of preterm infants to evaluate the effect of loss in weight z-score from birth to 36 weeks PMA greater than 1, using the Fenton preterm growth charts on their growth pattern until two years CA. Weight was measured at a standard time each day using an electronic scale (Kern MBC 15K2DM, Kern and Sohn, Balingen, Germany) or an incubator with adequate calibration (Babyleo TN500, Draeger, Lübeck, Germany), accurate to the nearest 5.0 g.

Infants were born in a tertiary referral neonatal unit at La Paz University Hospital (LPUH, Madrid, Spain) between January 2020 and December 2021. To assess growth trajectory, we collected weight, length, and head circumference after discharge at 3, 6, 12, and 24 months CA. Weight was measured accurately to the nearest 10 g (Seca electronic infant scale, Seca 375, Hamburg, Germany). Length was measured accurately to the nearest 0.1 cm (Seca infant length board, Seca 210, Hamburg, Germany). HC measures were taken with a non-stretchable tape measure to the nearest 0.1 cm.

The inclusion criteria were infants born at La Paz University Hospital, with a gestational age (GA) < 32 weeks and/or a birthweight < 1500 g (very low birthweight (VLBW) infants), who completed the follow-up at the neonatology outpatient clinic. Exclusion criteria were infants born with chromosomal or congenital anomalies who were transferred to another hospital, with a death and loss of follow-up after discharge. We considered the loss of follow-up when more than two visits after discharge were missing; infants who attended at least 2 out of 4 visits after discharge in the outpatient clinic up to 2 years CA. During the study period, 249 VLBW infants and/or GA < 32 weeks of gestation were born at La Paz University Hospital.

We divided the sample into two groups according to growth trajectory during the initial hospital stay: infants who experienced GF and infants who did not experience GF (NGF). GF is defined as a loss of 1 z-score in weight-for-age at discharge, at least 1 month after birth [16].

We used weight, length, and head circumference z-scores. When infants were <50 weeks of post-menstrual age (PMA) we used Fenton growth charts for preterm infants, and for the measurements at 3, 6, 12 and 24 months CA the WHO growth charts for infants < 2 years old were consulted through the WHO Anthro Survey Analyser and the WHO Anthro app, using the estimated due date as estimated birth date (40 weeks PMA). We compared each individual to their birth z-scores and used the 36-week PMA data to define the groups.

To assess growth rates, we calculated weight (g/kg/day), length (cm/week), and head circumference gain (cm/week) from discharge to 3, 6, 12, and 24 months CA. Short-term morbidity included BPD and LOS. BPD was defined as a requirement for supplemental oxygen at 36 weeks PMA [17]. LOS was defined as clinical findings with a positive blood culture. NEC diagnosis based on clinical signs and radiological findings—Bell’s stages 2 and 3—was considered definitive disease.

To assess neurodevelopment, Parent Report of Children’s Abilities-Revised (PARCA) and Bayley-III scores were used. PARCA is applied to infants born <32 weeks and/or a birthweight < 1500 g, and Bayley-III to every infant born <28 weeks and/or a birthweight < 1000. One infant may have both scores. The PARCA is a caregiver-completed questionnaire designed to assess cognitive and language development in children around 24 months in CA.

### 2.2. Primary Outcome

The main outcome of this study is to detect if preterm infants with GF at discharge had accelerated growth after discharge and were able to recover the birth z-scores of weight, length, and head circumference at 3, 6, 12, and 24 months CA, compared to infants without GF.

### 2.3. Secondary Outcomes

For the secondary outcome, we collected data from the PARCA and Bayley-III tests to assess neurodevelopment at 2 years CA and its relationship with GF.

### 2.4. Nutritional Protocol

International recommendations on early nutrition were followed, starting with parenteral nutrition immediately at birth until exclusive enteral nutrition (>120 mL/kg/day) was achieved. All enrolled infants received lipids with SMOFlipid (Fresenius Kabi AB, Uppsala, Sweden). Enteral nutrition was introduced in the first 24 h after birth. Advances in enteral feeding volumes were made based on the signs of tolerance: vomiting, abdominal distension, and gastric residuals. The goal of enteral nutrition was to reach 150 mL/kg/day using the mother’s own milk (OMM) or donor human milk (DHM) when OMM was unavailable. Standard fortification of human milk started when enteral feeding reached 100 mL/kg/day and continued until discharge.

### 2.5. Statistical Analysis

Quantitative variables were analyzed using Student’s T test comparison or U–Mann–Whitney test for non-parametric data and by X^2^ exact test or Fisher’s exact test for qualitative variables, depending on the number of observations. For the repeated measures analysis, the Skillings–Mack test was performed, with the Wilcoxon signed-rank test as a post hoc test. Normality was assessed using the Shapiro–Wilk test.

Missing values were considered missing completely at random (MCAR). We performed single mean imputation when missing data was <10% of the total cases. For those variables in which the missing data reached >10% of the cases, we performed a multiple imputation using multivariate normal regression, and to evaluate the imputation, we performed a visual inspection of kernel density estimates plots. The multiple imputation was used only for the regression analysis of the main outcome. After missing values imputation, regression models were used to compare the 2 year CA z-score with the birth z-score of weight, length, and HC. These models were adjusted by GA (continuous), sex (male/female), BPD (yes/no), and NEC. Estimated beta (β) coefficients ± standard error (SE) and associated *p*-values were extracted from the models. Results were considered statistically significant for *p*-value < 0.05.

All data analyses were performed using Stata/MP (version 17.0). For the repeated measures analysis, the st0167_1 package was installed in STATA. Plots were generated using R version 4.3.0 (21 April 2023) within RStudio (version 2023.06.0, Rstudio, Inc., Vienna, Austria) with ggplot2 and dplyr packages.

## 3. Results

### 3.1. Population

**Figure 1:**
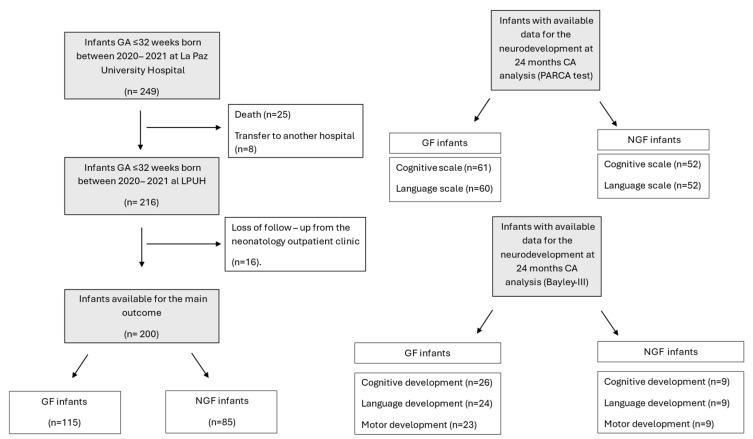
Flowchart diagram of the participant’s selection. GF = Growth Faltering; NGF = No Growth Faltering.

Two hundred preterm infants were included for the main outcome (Figure 1). Table 1 shows the characteristics of the participants. GA was lower in the GF group, with longer hospital stay and higher PMA at discharge. LOS and BPD incidence were higher in the GF group. NGF infants had a lower birthweight z-score and a higher proportion of SGA infants. Regarding nutrition, NGF had higher enteral intakes in volume at 28 days of life and at discharge. Hydrolyzed/elemental formula consumption at discharge was higher in the GF group. The Bayley-III test was available in 35 infants, and the PARCA in 113 infants at 2 years CA.

### 3.2. Anthropometric Differences in Both Groups Compared to Birth z-Score

**Figure 2:**
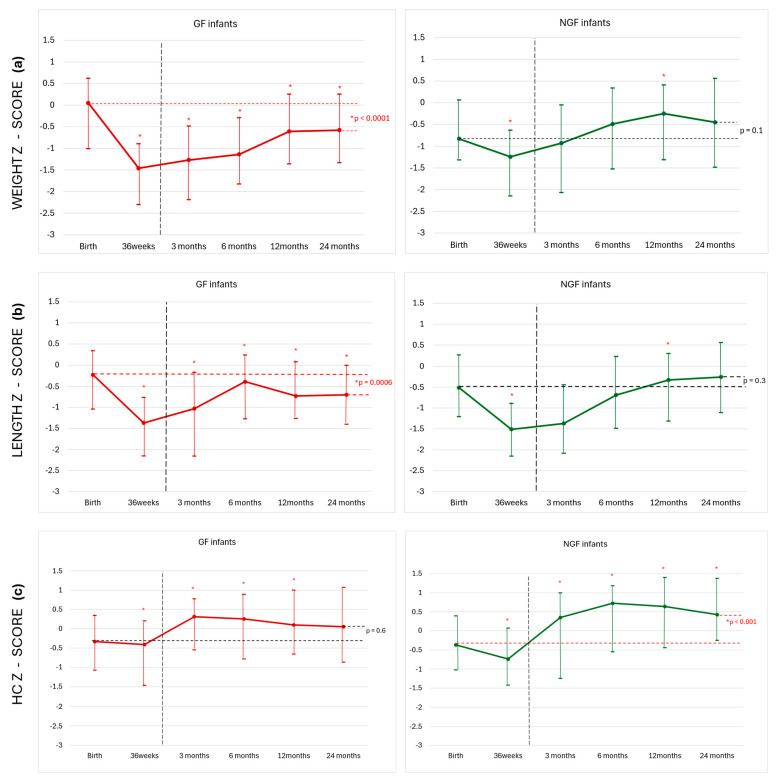
(**a**–**c**). Weight, length, head circumference, and z-score from birth to 2 years corrected age (CA) in both groups are represented with the median value. The Wilcoxon test was used as a post hoc analysis to analyze the differences between the different time points compared to birth in each group. Vertical line indicates the end of use of the Fenton charts; from 3 months, the CA WHO charts for infant growth were used. GF = Growth faltering; NGF = No growth faltering, HC = Head Circumference. * *p* > 0.05.

We performed a comparison of repeated measures of the z-scores of weight, length, and HC at 36 weeks, 3, 6, 12, and 24 months CA and the birth z-scores in both groups (Figure 2 and Supplementary Table S1).

Regarding weight (Figure 2a), the NFG group z-score was significantly lower at 36 PMA compared to birth (*p* < 0.001), at 1 year CA was significantly higher (*p* = 0.003), and by 2 years CA, there were no differences compared to the birth z-scores (−0.83 [−1.31; 0.06]). In the GF infants’ group, statistical differences were found at all times compared to birth. At 3 and 6 months, CA GF infants were still in z-score values lower than −1 point compared to the birth median value (0.05 [−1.01; 0.62]). At 12 and 24 months CA, they still had not regained the necessary weight to achieve birth z-score values.

Length z-score over time (Figure 2b) was similar in both groups, and at 2 years, CA remained significantly lower than at birth in the GF group. At 3 months, GF infants were still in z-score values lower than −1 point compared to the birth median value. Head circumference increased significantly in the NGF group compared to birth (*p* < 0.001) (Figure 2c). In infants with GF, the HC z-score at 2 years CA was similar to birth.

### 3.3. Anthropometric Measurements Differences Between Groups After Discharge

Regarding growth rates, weight gain in g/kg/day was not different between groups (Table 2). Length gain in cm/week was significantly higher at 3 months CA in the NGF group and at 12 months CA. HC gain was higher in the NGF group at 2 years old CA.

### 3.4. Multivariate Analysis of the 24-Month z-Score Compared to Birth

Regression models were used to assess the difference between the 2-year CA time point value and the birth z-score, adjusted by sex, NEC, BPD, and GA (Table 3).

After adjusting for relevant covariates, GF infants exhibited a significantly lower weight z-score at 2 years of age compared with their z-score at birth. In contrast, length z-scores did not demonstrate significant differences over time, and head circumference z-scores showed a slight increase.

In the group of NGF infants, no significant changes were observed in weight or length z-scores between birth and 2 years of age after adjustment for covariates. However, head circumference z-scores increased notably and reached statistical significance.

### 3.5. Neurodevelopment Differences Between Groups at 2 Years CA

- Unadjusted analysis.

The Bayley-III test was available for 35 infants. We found a significant difference in motor development, with a higher score in the NGF group (94 [88; 100] vs. 85 [79; 91], *p* = 0.03) (Table 4). In PARCA scales, a significant difference was found in the non-verbal cognitive scale, favoring the NGF group (84.5 [69.5; 91.5] vs. 88.5 [78.5; 96.5], *p* = 0.037) (Table 4).

b. Adjusted analysis.

After adjusting for sex, GA, LOS, NEC, and BPD, no effect of GF was observed in neurodevelopment. NEC significantly affected the language scale of the Bayley-III (β ± SE = −20 ± 7.6, *p* = 0.014), as well as both evaluated PARCA scales (language scale: β ± SE = −26.2 ± 8.5, *p* = 0.003; non-verbal cognitive scale: β ± SE = −43.9 ± 10.3, *p* < 0.0001). We also observed a negative effect of BPD on language development measured by Bayley-III (β ± SE = −10.4 ± 4.4, *p* = 0.025) and motor development (β ± SE = −11 ± 5, *p* = 0.04).

## 4. Discussion

GF infants do not recover birth weight z-score in the first two years of life. The weight z-score remains lower until six months CA in GF compared to NGF infants, and the length z-score remains lower until the first year of CA. HC z-score is lower at all time points in the GF group compared to the NGF infants. Our data shows similar weight gain from discharge to 2 years CA between infants who had GF and infants who left the hospital NGF. GF infants had significantly lower punctuation in the motor development test of the Bayley-III and in the language scale of PARCA at 2 years old CA before correction for confounding factors.

### 4.1. Risk of Malnutrition in Premature Infants

The most common markers of adequate nutritional status are serial measurements of anthropometrics (weight, length, and occipitofrontal circumference (OFC)). Ideally, postnatal growth studies should consider relationships with baseline variables [18] as has been performed in our study. We have considered in the multivariate regression morbidities and gestational age as confounding variables, and even then, differences with birthweight z-scores remain in the GF group. The secondary indicator of malnutrition is the decline in length-for-age z-score (>0.8) [19]. Our GF infants showed a higher decline in length z-score from birth to discharge compared to NGF.

Premature infants are at risk for developmental delays. Growth of premature infants in the first weeks has been associated with neurodevelopment [20,21]. Studies including weight status at 36 weeks are difficult to interpret because they did not account for the infant’s initial growth percentiles [22]. Postnatal GF has been reported as a poor predictor of neurodevelopmental outcomes [23]. Slower growth in length was associated with lower IQ scores at age 8 [20]. It has been previously described how HC growth is associated with neurodevelopment at 2 years CA [24] in preterm infants. To address this concern, we and others consider changes in weight, length, or OFC z-score as a better measure for adequate growth [25,26].

Our results are similar regarding differences in weight and length after 12 months of CA in the GF group to the data presented by Cooke et al. in infants who are more mature and with higher birth weight [27]. Our NGF infants present similar linear growth as reported recently in the experimental arm of a randomized controlled trial [28]. Strobet et al. reported that infants with head circumference GF at 2 years, defined as loss of z-score between 2 years and discharge greater than 0.8, had lower adjusted Bayley-III language scores (−4.0 [−8.0, −0.1]) compared to the normal head circumference growth cohort [29]. Other studies have shown that greater weight gain at term was associated with better 18-month neurodevelopmental outcomes [30]. Additionally, Ariel A Salas et al. [26], similarly to our results, associated a decline below −1.0 in weight z-score from birth to 36 weeks PMA with a higher risk of cognitive delay at 2 years of age (Bayley-III cognitive score < 85). Growth velocity during VLBW infants’ NICU hospitalization exerts a significant, and possibly independent, effect on neurodevelopmental and growth outcomes at 18 to 22 months’ CA [31]. In an observational study, an association between lower body weight at CA of 6, 12, and 24 months and worse neurodevelopmental outcomes among VLBW premature infants has been observed [21].

In a systematic review, which uses a more restrictive definition of GF (>0.8 difference in z-score in weight and length and >1 in HC), postnatal growth patterns in preterm infants were consistently associated with long-term neurocognitive and cardiometabolic outcomes, including obesity, hypertension, and insulin resistance [32]. The authors highlight that both early growth faltering and rapid catch-up growth may contribute to later health risk, while emphasizing the lack of high-quality interventional studies to define optimal growth trajectories. From a broader perspective, evidence across populations indicates that early growth faltering is associated with persistent adverse health and functional outcomes later in life, supporting the role of impaired early growth as an independent determinant of long-term risk [33]. More longitudinal studies are needed to determine the consequences for health of restricted growth during early life.

### 4.2. Premature Infants and Neurodevelopment

Comorbidities such as BPD may influence neurodevelopment at 2 years CA [34]. Additionally, NEC in preterm infants has been consistently associated with adverse neurodevelopmental outcomes by 2 years’ CA. Meta-analytic data indicate that NEC increases the risk of neurodevelopmental impairment, with an adjusted odds ratio around 1.89 (95% CI 1.46–2.46) versus non-NEC peers [35]. Moreover, in VLBW infants, surgical NEC (as opposed to medically managed NEC) has been linked to higher rates of cerebral palsy, lower psychomotor scores, and microcephaly at 24 months CA [36]. We showed the effect of growth during the initial stay on neurodevelopment, although this effect disappeared when morbidity was considered. Limited sample size may influence our results. Studies of the association between z-score after discharge of Extremely Low Birthweight infants and neurodevelopmental outcomes show mixed results [21,37].

Overall, growth differs between groups at 2 years—CA, depending on growth during hospital stay, independent of the comorbidities. Neurodevelopment at 2 years CA is mainly associated with NEC and BPD diagnoses.

### 4.3. Limitations

The main limitation of this retrospective study is that the feeding type after discharge is unknown; all infants received the same information from the outpatient clinic, and no intervention was performed. As our objective was to study the impact that GF during hospitalization may have on growth trajectories and neurodevelopment at 2 years CA, we consider our results to be relevant and consistent with the current evidence, although the NGF sample for the Bayley-III was small. This evaluation was only performed in infants below 28 weeks GA and/or birthweight less than 1000 g. Finally, the use of 1SD loss for defining growth failure can be discussed.

## 5. Conclusions

In this cohort study, we identified that the GF of infants born earlier than 32 weeks GA and/or a birthweight < 1500 g is associated with growth trajectories up to 2 years CA. The nutritional requirements and the management of infants during hospital stay should be revisited if they have growth failure, since the loss of 1 point of weight z-score seems to have long-term effects. NEC and BPD may be associated with a higher risk of adverse neurodevelopmental outcomes at 2 years CA.

## Figures and Tables

**Table 1:** Basic Characteristics of the study population.

	GF During Hospital Stay		
	Yes (n = 115)	No (n = 85)	*p*
Boys	54 (46.9)	48 (56.5)	0.2
Birth to discharge (days)	60 [40; 82]	43 [33; 50]	<0.0001
Singleton pregnancy	47 (40.9)	28 (32.9)	0.2
Mother’s age at childbirth, years	35 [31; 37]	35 [31; 38]	0.4
GA at birth (Weeks + days)	29 + 0 ± 2.4	30 + 5 ± 2.3	<0.0001
Birth weight (grams ± SD)	1149 ± 327	1271 ± 313	0.008
Weight z-score (SD) at birth	−0.16 ± 1	−0.75 ± 0.97	0.0001
Birth length (cm)	36.5 [34.5; 39]	39 [36; 40.5]	0.001
Length z-score (SD) at birth	−0.3 [−0.9; 0.3]	−0.52 [−1.5; 0.1]	0.06
Birth HC (cm)	26 [24; 28]	28 [26; 29]	<0.0001
HC z-score (SD) at birth	−0.5 ± 1.11	−0.4 ± 1.2	0.9
SGA n (%)	20 (17.4)	25 (29.4)	0.04
PMA at discharge (Weeks + days)	37 + 2 [36 + 2; 39 + 0]	36 + 6 [35 + 6; 38 + 0]	0.02
Weight at discharge (grams)	2270 [2040; 2560]	2295 [2085; 2520]	0.8
Weight z-score (SDS) at discharge	−1.5 [−2.5; −0.8]	−1.2 [−2.1; −0.6]	0.04
Length at discharge (cm)	44.5 [43.5; 46]	44 [43; 45.5]	0.3
Length z-score at discharge	−1.4 [−2.3; −0.83]	−1.2 [−2.1; −0.7]	0.4
Head circumference (cm) at discharge	32 [31.5; 33]	32.5 [31.5; 33.5]	0.2
Head circumference z-score (SDS) at discharge	−0.8 ± 0.9	−0.3 ± 1.0	0.001
Fortified at discharge	70 (60.9)	64 (75.3)	0.03
Hydrolyzed formula at discharge	14 (12.2)	1 (1.2)	0.004
NEC	9 (7.8)	2 (2.3)	0.09
Bronchopulmonary dysplasia	35 (30.4)	11 (12.9)	0.004
Sepsis	47 (40.9)	12 (14.1)	<0.0001

Values expressed as mean ± standard deviation (SD) or 50th percentile and quartiles (Q1; Q3) in quantitative variables, depending on the distribution of the data; and sample size (n) and relative frequency (%) in qualitative variables. Quantitative variables were analyzed using Student’s *t*-test comparison or the U–Mann–Whitney test for non-parametric data. Qualitative variables differences were analyzed using X^2^ Pearson’s test. SD = Standard Deviation; GA = Gestational Age; HC = Head Circumference; GF = Growth faltering; PMA = Post-menstrual Age; SGA: Small for gestational age; NEC: Necrotizing Enterocolitis. GA and PMA deviation were calculated using the decimal GA and PMA.

**Table 2:** Growth rates comparison between groups from discharge.

	Weight Gain (g/kg/day)		
	GF (n = 115)	NGF (n = 85)	*p*
3 months CA	7.04 ± 1.39 (104)	7.21 ± 1.22 (74)	0.4
6 months CA	4.74 [4.43; 5.10] (93)	4.75 [4.34; 5.28] (71)	0.7
12 months CA	2.96 [2.73; 3.22] (89)	3.02 [2.8; 3.25] (72)	0.5
24 months CA	1.74 [1.61; 1.83] (100)	1.73 [1.61; 1.85] (78)	0.9
	**Length Gain (cm/week)**		
	GF (n = 115)	NGF (n = 85)	
3 months CA	0.85 [0.75; 0.95] (104)	0.91 [0.83; 1] (74)	0.01
6 months CA	0.69 [0.64; 0.76] (90)	0.73 [0.66; 0.77] (71)	0.6
12 months CA	0.51 [0.47; 0.55] (85)	0.54 [0.5; 0.57] (72)	0.04
24 months CA	0.38 [0.35; 0.4] (100)	0.38 [0.34; 0.4] (77)	0.7
	**HC Gain (cm/week)**		
	GF (n = 115)	NGF (n = 85)	
3 months CA	0.48 [0.44; 0.54] (87)	0.5 [0.45; 0.55] (70)	0.1
6 months CA	0.87 [0.79; 0.96] (87)	0.85 [0.80; 0.95] (62)	0.8
12 months CA	0.24 [0.22; 0.26] (84)	0.25 [0.23; 0.27] (72)	0.1
24 months CA	0.128 [0.12; 0.14] (99)	0.146 [0.13; 0.16] (77)	0.04

Values expressed as 50th percentile and quartiles (Q1; Q3) in quantitative variables. Data was analyzed using *the U–Mann–Whitney* test for non-parametric data. CA = Corrected age; HC = Head Circumference; GF = Growth faltering; NGF = No growth faltering.

**Table 3:** Regression models comparing the 2-year-CA z-score of weight, length, and HC with the birth z-score.

**Weight**	**z-score 24 months CA**	
	β ± SE	*p*-value
GF (n = 115)	−0.5 ± 0.2	<0.0001
NGF (n = 85)	0.3 ± 0.2	0.07
**Length**	**z-score 24 months CA**	
	β ± SE	*p*-value
GF (n = 115)	−0.15 ± 0.2	0.5
NGF (n = 85)	0.1 ± 0.2	0.7
**HC**	**z-score 24 months CA**	
	β ± SE	*p*-value
GF (n = 115)	0.3 ± 0.2	0.04
NGF (n = 85)	1.4 ± 0.5	0.003

Data shows estimated beta (β) coefficients ± standard error (SE) and *p*-value. Birthweight, birth length, and birth HC z-score were considered the reference. Models were adjusted by sex, sepsis, and bronchopulmonary dysplasia during hospital stay and gestational age. CA = Corrected age; HC = Head Circumference; GF = Growth faltering; NGF = No growth faltering.

**Table 4:** Differences in neurodevelopment scales between groups. (**a**) Bayley-III scores. (**b**) PARCA scores.

(a)			
	**Cognitive Development**		
	GF (n = 26)	NGF (n = 9)	*p*
Score	95 [90; 100]	100 [95; 100]	0.25
	**Language Development**		
	GF (n = 24)	NGF (n = 9)	
Score	90.5 [81; 100]	94 [94; 100]	0.17
	**Motor Development**		
	GF (n = 23)	NGF (n = 9)	
Score	85 [79; 91]	94 [88; 100]	0.033
(**b**)			
	**Non-Verbal Cognitive Scale**		
	GF (n = 61)	NGF (n = 52)	*p*
Score	87 [78; 97]	92 [81; 102]	0.076
	**Language Scale**		
	GF (n = 60)	NGF (n = 52)	
Score	84.5 [69.5; 91.5]	88.5 [78.5; 96.5]	0.037

Values expressed as 50th percentile and quartiles (Q1; Q3). Data was analyzed using the U–Mann–Whitney test for non-parametric data. GF = Growth faltering; NGF = No growth faltering.

## Data Availability

The data that support the findings of this study are not publicly available due to containing information that could compromise the privacy of research participants, but are available from the corresponding author [A.W.C.] upon request.

## Supplementary Materials

The following supporting information can be downloaded at: https://www.mdpi.com/article/10.3390/nu18010125/s1, Supplementary Table S1: Differences in z-score median values between groups at 3-, 6-, 12-, and 24 months CA.

## Abbreviations

The following abbreviations are used in this manuscript:

PMA	Post Menstrual Age
GF	Growth Faltering
UK	United Kingdom
WHO	World Heatlh Organization
BPD	Bronchopulmonary Dysplasia
LOS	Late onset sepsis
NEC	Necrotizing Enterocolitis
GA	Gestational Age
NICU	Neonatal Intensive Care Unit
CA	Corrected Age
VLBW	Very Low Birth Weight
PARCA	Parent Report of Children’s Abilities-Revised
DHM	Donor’s Human Milk
OMM	Own’s Mother Milk
OFC	Occipitofrontal Circumference

## References

[1] Toftlund L.H., Halken S., Agertoft L., Zachariassen G. (2018). Catch-Up Growth, Rapid Weight Growth, and Continuous Growth from Birth to 6 Years of Age in Very-Preterm-Born Children. Neonatology.

[2] Rochow N., Raja P., Liu K., Fenton T., Landau-Crangle E., Göttler S., Jahn A., Lee S., Seigel S., Campbell D. (2016). Physiological adjustment to postnatal growth trajectories in healthy preterm infants. Pediatr. Res..

[3] Clark R.H., Thomas P., Peabody J. (2003). Extrauterine growth restriction remains a serious problem in prematurely born neonates. Pediatrics.

[4] González-García L., Mantecón-Fernández L., Suárez-Rodríguez M., Arias-Llorente R., Lareu-Vidal S., Ibáñez-Fernández A., Caunedo-Jiménez M., González-López C., Fernández-Morán E., Fernández-Colomer B. (2022). Postnatal Growth Faltering: Growth and Height Improvement at Two Years in Children with Very Low Birth Weight between 2002–2017. Children.

[5] Murray Y.L., Paul I.M., Miller J.R., Thrash S.Z., Kaiser J.R. (2021). Variability in the use of growth curves between preterm and term infants in NICUs and newborn nurseries. Pediatr. Res..

[6] (2017). National Guideline Alliance (UK). Faltering Growth—Recognition and Management.

[7] De Onis M., Blössner M. (1997). WHO Global Database on Child Growth and Malnutrition.

[8] McLeod G., Sherriff J. (2007). Preventing postnatal growth failure—The significance of feeding when the preterm infant is clinically stable. Early Hum. Dev..

[9] Shlomai N.O., Reichman B., Zaslavsky-Paltiel I., Lerner-Geva L., Eventov-Friedman S. (2022). Neonatal morbidities and postnatal growth failure in very low birth weight, very preterm infants. Acta Paediatr. Int. J. Paediatr..

[10] Flannery D.D., Jensen E.A., Tomlinson L.A., Yu Y., Ying G.S., Binenbaum G. (2021). Poor postnatal weight growth is a late finding after sepsis in very preterm infants. Arch. Dis. Child. Fetal Neonatal Ed..

[11] Speer A.L., Lally K.P., Pedroza C., Zhang Y., Poindexter B.B., Chwals W.J., Hintz S.R., Besner G.E., Stevenson D.K., Ohls R.K. (2024). Surgical Necrotizing Enterocolitis and Spontaneous Intestinal Perforation Lead to Severe Growth Failure in Infants: A Preplanned Secondary Analysis of the Necrotizing Enterocolitis Surgery Trial. Ann. Surg..

[12] Zozaya C., Avila-Alvarez A., Arruza L., Rodrigo F.G.-M., Fernandez-Perez C., Castro A., Cuesta M.T., Vacas B., Couce M.L., Torres M.V. (2019). The Effect of Morbidity and Sex on Postnatal Growth of Very Preterm Infants: A Multicenter Cohort Study. Neonatology.

[13] Embleton N.E., Pang N., Cooke R.J. (2001). Postnatal malnutrition and growth retardation: An inevitable consequence of current recommendations in preterm infants?. Pediatrics.

[14] Ramel S.E., Demerath E.W., Gray H.L., Younge N., Boys C., Georgieff M.K. (2012). The relationship of poor linear growth velocity with neonatal illness and two-year neurodevelopment in preterm infants. Neonatology.

[15] Hamatschek C., Yousuf E.I., Möllers L.S., So H.Y., Morrison K.M., Fusch C., Rochow N. (2020). Fat and Fat-Free Mass of Preterm and Term Infants from Birth to Six Months: A Review of Current Evidence. Nutrients.

[16] Cooke R., Goulet O., Huysentruyt K., Joosten K., Khadilkar A.V., Mao M., Meyer R., Prentice A.M., Singhal A. (2023). Catch-Up Growth in Infants and Young Children With Faltering Growth: Expert Opinion to Guide General Clinicians. J. Pediatr. Gastroenterol. Nutr..

[17] Jobe A.H., Bancalari E. (2001). Bronchopulmonary dysplasia. Am. J. Respir. Crit. Care Med..

[18] Fenton T.R., Cormack B., Goldberg D., Nasser R., Alshaikh B., Eliasziw M., Hay W.W., Hoyos A., Anderson D., Bloomfield F. (2020). Extrauterine growth restriction’ and ‘postnatal growth failure’ are misnomers for preterm infants. J. Perinatol..

[19] Goldberg D.L., Becker P.J., Brigham K., Carlson S., Fleck L., Gollins L., Sandrock M., Fullmer M., Van Poots H.A. (2018). Identifying Malnutrition in Preterm and Neonatal Populations: Recommended Indicators. J. Acad. Nutr. Diet..

[20] Brinkis R., Albertsson-Wikland K., Tamelienė R., Aldakauskienė I., Rimdeikienė I., Marmienė V., Šmigelskas K., Verkauskienė R. (2022). Impact of Early Nutrient Intake and First Year Growth on Neurodevelopment of Very Low Birth Weight Newborns. Nutrients.

[21] Hsu C.T., Chen C.H., Lin M.C., Wang T.M., Hsu Y.C. (2018). Post-discharge body weight and neurodevelopmental outcomes among very low birth weight infants in Taiwan: A nationwide cohort study. PLoS ONE.

[22] Fenton T.R., Kim J.H. (2013). A systematic review and meta-analysis to revise the Fenton growth chart for preterm infants. BMC Pediatr.

[23] Nyakotey D.A., Clarke A.M., Cormack B.E., Bloomfield F.H., Harding J.E. (2024). Postnatal growth and neurodevelopment at 2 years’ corrected age in extremely low birthweight infants. Pediatr. Res..

[24] Sicard M., Nusinovici S., Hanf M., Muller J.-B., Guellec I., Ancel P.-Y., Gascoin G., Rozé J.-C., Flamant C. (2017). Fetal and Postnatal Head Circumference Growth: Synergetic Factors for Neurodevelopmental Outcome at 2 Years of Age for Preterm Infants. Neonatology.

[25] Zozaya C., Díaz C., De Pipaón M.S. (2018). How Should We Define Postnatal Growth Restriction in Preterm Infants?. Neonatology.

[26] Salas A.A., Bhatia A., Carlo W.A. (2020). Postnatal growth of preterm infants 24 to 26 weeks of gestation and cognitive outcomes at 2 years of age. Pediatr. Res..

[27] Cooke R.J., Embleton N.D., Griffin I.J., Wells J.C., McCormick K.P. (2001). Feeding preterm infants after hospital discharge: Growth and development at 18 months of age. Pediatr. Res..

[28] Rossholt M.E., Bratlie M., Wendel K., Aas M.F., Gunnarsdottir G., Fugelseth D., Pripp A.H., Domellöf M., Størdal K., Stiris T. (2023). Effect of arachidonic and docosahexaenoic acid supplementation on quality of growth in preterm infants: A secondary analysis of a randomized controlled trial. Clin. Nutr..

[29] Strobel K.M., Wood T.R., Valentine G.C., German K.R., Gogcu S., Hendrixson D.T., Kolnik S.E., Law J.B., Mayock D.E., Comstock B.A. (2024). Contemporary definitions of infant growth failure and neurodevelopmental and behavioral outcomes in extremely premature infants at two years of age. J. Perinatol..

[30] Belfort M.B., Rifas-Shiman S.L., Sullivan T., Collins C.T., McPhee A.J., Ryan P., Kleinman K.P., Gillman M.W., Gibson R.A., Makrides M. (2011). Infant growth before and after term: Effects on neurodevelopment in preterm infants. Pediatrics.

[31] Ehrenkranz R.A., Dusick A.M., Vohr B.R., Wright L.L., Wrage L.A., Poole W.K. (2006). Growth in the neonatal intensive care unit influences neurodevelopmental and growth outcomes of extremely low birth weight infants. Pediatrics.

[32] Ong K.K., Kennedy K., Castañeda-Gutiérrez E., Forsyth S., Godfrey K.M., Koletzko B., Latulippe M.E., Ozanne S.E., Rueda R., Schoemaker M.H. (2015). Postnatal growth in preterm infants and later health outcomes: A systematic review. Acta Paediatr..

[33] Mertens A., Benjamin-Chung J., Colford J.M., Coyle J., van der Laan M.J., E Hubbard A., Rosete S., Malenica I., Hejazi N., Sofrygin O. (2023). Causes and consequences of child growth faltering in low-resource settings. Nature.

[34] DeMauro S.B. (2021). Neurodevelopmental outcomes of infants with bronchopulmonary dysplasia. Pediatr. Pulmonol..

[35] Wang Y., Liu S., Lu M., Huang T., Huang L. (2024). Neurodevelopmental outcomes of preterm with necrotizing enterocolitis: A systematic review and meta-analysis. Eur. J. Pediatr..

[36] Martin C.R., Dammann O., Allred E.N., Patel S., O’Shea T.M., Kuban K.C., Leviton A. (2010). Neurodevelopment of extremely preterm infants who had necrotizing enterocolitis with or without late bacteremia. J. Pediatr..

[37] O’Shea T.M., Register H.M., Yi J.X., Jensen E.T., Joseph R.M., Kuban K.C., Frazier J.A., Washburn L., Belfort M., South A.M. (2023). Growth During Infancy After Extremely Preterm Birth: Associations with Later Neurodevelopmental and Health Outcomes. J. Pediatr..

