# Supplementary material for: Assessment of Post-Discharge Growth Pattern After Initial Growth Faltering and Its Association with the Neurodevelopment Status in Preterm Infants: A Cohort Study
Source: Nutrients. 2025 Dec 30;18(1):125. doi: 10.3390/nu18010125 (PMC12787592; doi:10.3390/nu18010125)
Supplement: Supplementary file 1 [file nutrients-18-00125-s001.zip › nutrients-4052940-supplementary.pdf]

Supplementary Table S1. Differences in z-score median values between groups at 3-, 6-, 12- and 24-months CA.

Median weight z-score values			
	GF (n=115)	NGF (n=85)	***p***
3 months CA	-1.27 [-2.18; -0.49] (104)	-0.93 [-1.69; -0.09] (73)	*
6 months CA	-1.14 [-1.82; -0.37] (93)	-0.49 [-1.52; 0.32] (70)	**
12 months CA	-0.61 [-1.31; 0.24] (89)	-0.25 [-1.31; 0.41] (71)	
24 months CA	-0.58 [-1.33; 0.18] (99)	-0.45 [-1.47; 0.55] (77)	
Median length z-score values			
	GF (n=115)	NGF (n=85)	*p*
3 months CA	-1.3 [-2.16; -0.57] (104)	-1.01 [-1.98; 0.11] (73)	*
6 months CA	-0.7 [-1.68; -0.08] (90)	-0.21 [-0.96; 0.42] (69)	**
12 months CA	-0.76 [-1,58; 0.09] (85)	-0.35 [-1.12; 0.33] (71)	*
24 months CA	-0.57 [-1.38; 0.08] (100)	-0.42 [-1.17; 0.35] (76)	
Median HC z-score values			
	GF (n=115)	NGF (n=85)	*p*
3 months CA	-0.1 [-1.03; 0.62] (103)	0.61 [-0.25; 1.32] (73)	***
6 months CA	-0.1 [-0.96; 0.89] (87)	0.63 [0.2; 1.18] (70)	***
12 months CA	0.005 [-1.0; 1.02] (84)	0.61 [-0.06; 1.45] (72)	**
24 months CA	-0.13 [-0.99; 0.58] (99)	0.69 [-0.16; 1.38] (77)	***

Values expressed as 50th percentile and quartiles (Q1; Q3) in quantitative variables. Data was analyzed using *U-Mann Whitney* test for non-parametric data (*p < 0.05; **p < 0.01; ***p < 0.001). CA = Corrected age; HC = Head Circumference; GF = Growth faltering; NGF = No growth faltering.
